# DRAM is Involved in Hypoxia/Ischemia-Induced Autophagic Apoptosis in Hepatocytes

**DOI:** 10.14336/AD.2018.0210

**Published:** 2019-02-01

**Authors:** Jianji Xu, Yunjin Zang, Dongjie Liu, Tongwang Yang, Jieling Wang, Yanjun Wang, Xiaoni Liu, Dexi Chen

**Affiliations:** ^1^Beijing You’an Hospital Affiliated with Capital Medical University, Beijing 100069, China; ^2^Beijing Institute of Hepatology, Capital Medical University, Beijing 100069, China; ^3^Organ Transplantation Center, The Affiliated Hospital of Qingdao University, Qingdao 266000, China; ^4^The Beijing Precision Medicine and Transformation Engineering Technology Research Center of Hepatitis and Liver Cancer, Beijing 100069, China

**Keywords:** DRAM, autophagy, apoptosis, hypoxia/ischemia, hepatocyte

## Abstract

Liver hypoxia/ischemia injury leads to acute liver injury, delayed graft dysfunction, and failure during liver transplantation. Previous studies showed that autophagy is involved in liver hypoxia/ischemia injury. Our and others’ studies have found that the damage-regulated autophagy modulator (DRAM) could induce the autophagic apoptosis. However, the role of DRAM regulating autophagy in liver hypoxia/ischemia injury remains unclear. The aim of this study was to determine whether DRAM is involved in oxygen-glucose deprivation (OGD)-induced hepatocyte autophagic apoptosis. Normal hepatocytes (HL-7702) were treated with OGD while Balb/c mice underwent surgery to induce 70% liver ischemia. To evaluate the role of DRAM in hypoxia/ischemia-induced hepatic injury, DRAM siRNA was used to knockdown DRAM expression in cultured hepatocytes and a recombinant adenovirus vector expressing DRAM was used to overexpress DRAM in cultured hepatocytes *in vitro* and in the liver *in vivo*. Hepatic injury was analyzed by histopathological methods and measurement of hepatocyte enzyme release. Cell apoptosis was analyzed by flow cytometry and TUNEL staining. Several autophagic biomarkers were observed by western blot analysis. OGD and 70% hepatic ischemia significantly induced cell autophagy, apoptosis and DRAM expression in hepatocytes *in vitro* and *in vivo*. OGD-induced autophagic apoptosis was inhibited by 3-Methyladenine (3-MA). OGD-induced injury and autophagy in HL-7702 cells were significantly attenuated by DRAM knockdown but aggravated by DRAM overexpression *in vitro*. Similarly, DRAM overexpression increased ischemia-induced liver injury and hepatic apoptosis *in vivo*. Our data demonstrate that hypoxia/ischemia induces hepatic injury through a DRAM-dependent autophagic apoptosis pathway. These data also suggest that DRAM plays an important role in ischemia-induced liver injury and hepatocyte apoptosis.

Liver ischemia/reperfusion injury is a leading cause of acute liver injury and delayed graft dysfunction and failure after liver transplantation in which there are no effective strategies to avoid this occurrence in the clinic. Liver ischemia, resulting in oxygen and nutrient deprivation, is characterized by the depletion of ATP. Subsequent changes in ion influx, perturbation of calcium homeostasis, and cell swelling may cause cell death and necrosis [1, 2]. The liver is one of the more vulnerable organs that experience hypoxia/ischemia injury in the clinic [3]. Therefore, to improve organ viability, finding a strategy to reduce liver hypoxia/ischemia injury is very urgent [4]. OGD is a frequently used model for studying ischemia *in vitro* [5-7], and OGD-induced hepatocyte injury can be used to mimic donor liver ischemic injury [8].

Autophagy is a regulated process of the cells by which unnecessary or dysfunctional components such as organelles and proteins are delivered to lysosomes for degradation, which is critical for cell survival, differentiation, and metabolism [9, 10]. However, immoderate activation of the autophagic pathway can also result in functional organelles being attacked and devoured [11], creating massive autophagic membrane structures such as autophagic vacuoles, phagophores, and autophagosomes in the dying cell [12]. Autophagy can be activated by starvation, hypoxia, and ischemia [13]. Light chain 3 (LC3), an autophagy marker protein, participates in autophagosome formation via converting cytosolic LC3-I to membrane-bound LC3-II [14]. Hence, the level of LC3-II reflects autophagy in the cell to a certain extent. The nucleoporin p62 complex binds to autophagy regulator autophagy-related protein 8 (Atg8)/LC3 in the LC3-interacting region (LIR). p62 is an autophagy substrate that can be used as a reporter of autophagy activity [15]. In most cases, the liver transplantation donor undergoes Ischemia/Reperfusion injury, however, the role of immoderate activation of the autophagy in liver graft dysfunction and failure is unclear.

DRAM is a lysosomal protein that contributes to p53-regulated autophagy induction [16]. Our previous study found that starvation-induced DRAM expression and DRAM-mediated autophagic apoptosis was observed in normal hepatocytes [17]. However, the effect of DRAM-mediated autophagy on liver ischemia/reperfusion injury has not been well determined. In this study, DRAM-associated autophagy and cell death were identified in cells treated with OGD *in vitro* and in a mouse model of 70% liver ischemia *in vivo*.

## MATERIALS AND METHODS

### Cell culture and OGD treatment

A human normal liver cell line HL-7702 was cultured in Dulbecco’s modified Eagle’s medium (Invitrogen, Carlsbad, CA, USA) supplemented with 10% fetal bovine serum, penicillin (100U/ml) and streptomycin (0.1 mg/ml) (Gibco, Grand Island, NY, USA). The cells were maintained at 37°C in a 5% CO2 humidified incubator. The OGD model was performed as previously described [5-7]. Briefly, the HL-7702 cells were washed twice with Phosphate Buffered Saline (PBS) and cultured with glucose-free Earle’s balanced salt solution then transferred to an anaerobic chamber containing 95% N2 and 5% CO2 at 37°C for various time intervals. Control group cells were treated identically without undergoing OGD conditions.

**Figure 1.**
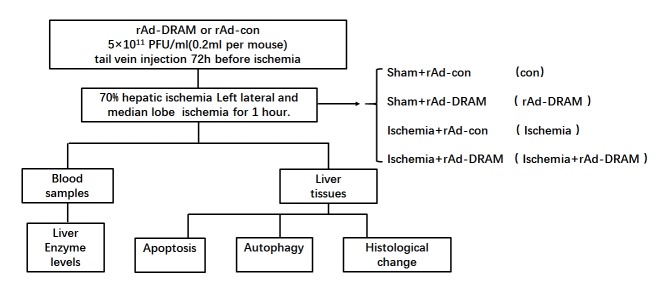
Experimental protocol for the study.

### Mice and 70% hepatic ischemia

A 70% hepatic ischemia mouse model was used to mimic liver hypoxia/ischemia injury *in vivo* [18, 19]. Male Balb/c mice (8-12 weeks old obtained from the Academy of Military Medical Sciences, China) were randomly divided into four groups of 6 animals each (Fig. 1). The mice were anesthetized with 30mg/kg sodium pentobarbital (Nembutal, St Louis, MO, USA) via intraperitoneal injection. After laparotomy, a vascular clip (Shanhe, Shanghai, China) was placed across the hepatic artery, portal vein and bile duct above the branching to the left lateral and median lobe for 1 hour. The DRAM-overexpressed group received rAd-DRAM (5×10^10^ PFU/ml, 0.2ml per mouse) while control animals received rAd-control (empty viral vector, 5×10^10^ PFU/ml, 0.2ml per mouse) via tail vein 72h before liver ischemia surgery (Fig. 1).

### Lactate dehydrogenase (LDH) assay

Cell injury or cell membrane permeability was also assessed using a lactate dehydrogenase (LDH) kit (Beyotime Science, Beijing, China). LDH levels in the cell supernatant were assessed according to the manufacturer’s protocol (Thermo MULTISKAN GO, Japan).

### Measurement of fluorescent LC3 puncta

HL-7702 cells were transfected with Ad-GFP-LC3 (5×10^7^ PFU/ml). Fresh media was changed two hours after transfection and the cells were further cultured for 46 hours. The cells underwent OGD treatment as described above and were then washed twice with PBS and fixed with 4% paraformaldehyde. GFP fluorescent and DAPI nuclear staining were observed under a confocal microscope (Leica, Solms, Germany).

### DRAM overexpression with rAd-DRAM and knockdown with DRAM siRNA

Purified recombinant adenovirus expressing DRAM (rAd-DRAM, 5×10^10^ PFU/ml) and control (rAd-control, 5×10^10^ PFU/ml) were purchased from Heyuan BioTech. Inc., Shanghai, China. The adenoviruses were stored in PBS containing 10% glycerol at -80°C. Before transfection, the adenovirus was diluted to the dose specified for each experimental group. DRAM siRNA (si-DRAM) were obtained from Crighton et al [12] and transfected into cells using Lipofectamine® 2000 (Thermo Fisher Scientific, Inc., Rockford, IL, USA) according to the manufacturer’s instruction. After transfection, cells were used for OGD treatment.

### Cells apoptosis assay

HL-7702 cells were infected with rAd-control and rAd-DRAM for 48h before OGD treatment. After OGD, the HL-7702 cells were washed twice with cold PBS, digested and resuspended in propidium iodide (PI) and annexin V binding buffer (Southern Biotech, Birmingham, USA). Cell apoptosis was analyzed by flow cytometry (BD FACSCanto II, Becton Dickinson, USA).

### Immunoblot assay

Cell lysates were subjected to western blot analysis as previously described [17]. Briefly, total cellular lysates were separated on 10-15% SDS-PAGE gels and transferred to a PVDF membrane. The membrane was then blocked with 5% non-fat milk and probed sequentially with specific primary antibodies and horseradish peroxidase-conjugated secondary antibodies. The detection of specific proteins on the blots was achieved with enhanced chemiluminescence (Pierce Super Signal, Thermo Fisher Scientific Inc., Rockford, IL, USA), and images were captured on X-ray films. The primary antibodies and dilutions used are listed as follows: p62 (1:1000), PARP (1:1000), p53 (Abcam, 1:1000) (Cell Signaling Technology, Danvers, USA). DRAM (1:1000) (Sigma, MO, USA). LC3B (1:1000), GAPDH (1:1000) (Cambridge, MA, USA).

### TUNEL staining

Frozen sections of liver tissue (5 μm) were subjected to TUNEL staining using a commercially available kit (In Situ Cell Death Detection kit, Roche-Boehringer, Mannheim, Germany).

### Serum ALT and AST measurements

The serum levels of alanine aminotransferase (ALT) and aspartate aminotransferase (AST) were measured with an AU5400 automated chemical analyzer (Olympus, Tokyo, Japan).

### Histopathology

Liver specimens were fixed with 4% paraformaldehyde and then embedded in paraffin. The specimens were sectioned at a thickness of 5 μm and stained with hematoxylin and eosin (HE) for histopathologic analysis by light microscopy. The severity of hypoxia/ischemia was graded blindly using modified Suzuki’s classification [20]. Sinusoidal congestion, hepatocyte necrosis, and vacuolization degeneration were graded on a scale of 0 to 4. No vacuolization, congestion, or necrosis were given a score of 0, whereas severe congestion/vacuolization and >60% lobular necrosis were given a value of 4.

### Statistical analyses

Data were expressed as the mean ± SD from at least three independent experiments. The difference between groups was compared using a one-way analysis of variance (ANOVA) using Prism 6 (GraphPad Software, La Jolla, CA). A value of p<0.05 was considered statistically significant.

## RESULTS

### ODG treatment induced activation of autophagy and increased apoptosis in HL-7702 cells

**Figure 2.**
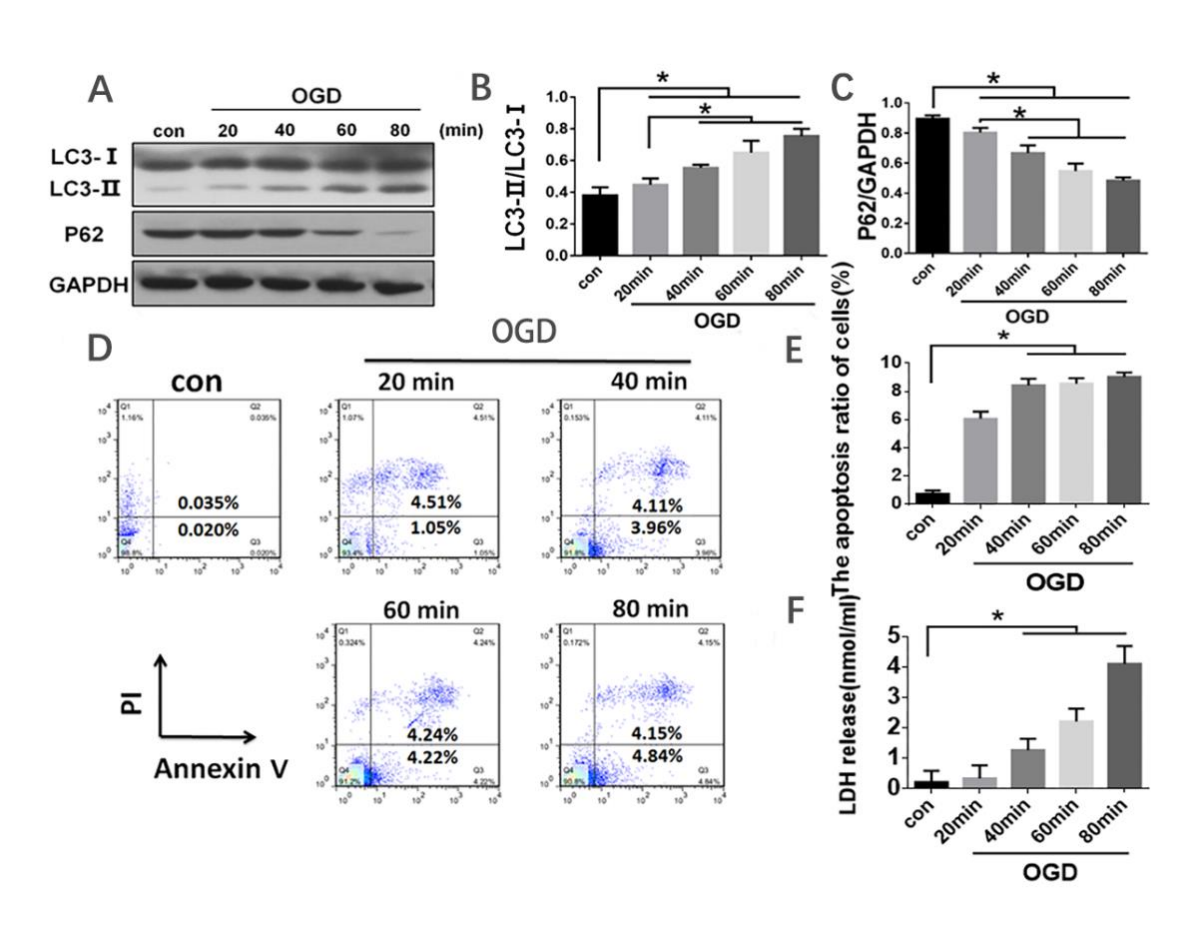
**ODG treatment induced activation of autophagy and apoptosis in HL-7702 cells**. The cells were treated with oxygen-glucose deprivation for 20 min, 40 min, 60 min, and 80 min, and the supernatant was collected for indicated experiments thereafter. (A) Western blot analysis was performed with antibodies against LC3 and p62 to detect autophagy. GAPDH was used as a loading control. (B and C) Quantitative densitometry scan results from (A). (D) Cells were stained with Annexin V/PI and analyzed by flow cytometry. (E) Apoptosis ratio of cells from (D). (F) Cell death was assessed by LDH release into the supernatant. Data in graph A,C,E and F were presented as mean ± SD from three independent experiments. *p<0.05.

HL-7702 were cultured with oxygen and glucose-free EBSS medium in a tri-gas incubator for 20 min, 40 min, 60 min, and 80 min. To determine whether autophagy was induced by OGD, LC3 and p62 expression levels were measured with western blot. OGD significantly increased LC3 II/I ratio in a time-dependent manner. OGD decreased p62 expression level as early as 20 minutes (Fig. 2A-C). To examine the events of cell death and apoptosis following OGD, cells were analyzed by annexin Ⅴ/PI staining and LDH release. The results showed that apoptosis and cell death in HL-7702 hepatocytes with OGD treatment increased greatly compared to the cells without OGD (Fig. 2D-F). We chose a 40 min OGD period for our following studies because the apoptotic rate in this time point increased significantly compared to 20 min OGD, and there were no significant changes between 40 min, 60 min, and 80 min OGD. Taken together, these data indicate that OGD treatment induced cell autophagy and apoptosis in HL-7702 cells.

**Figure 3.**
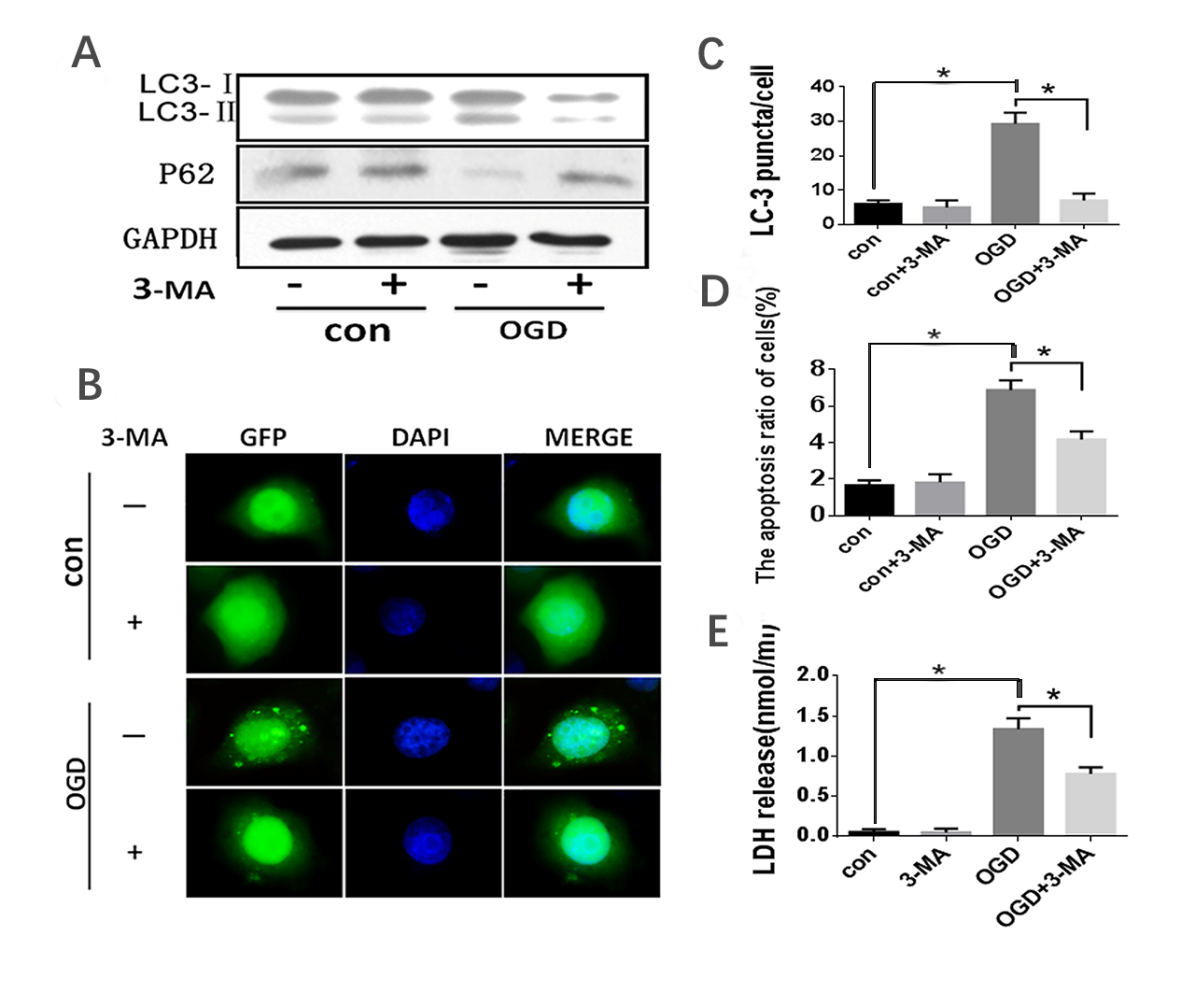
**OGD treatment induced autophagy activation in HL-7702 cells**. HL-7702 cells underwent OGD for 40 minutes with or without pre-treatment of 5 mM 3-MA for 3 hours. (A) Western blot analysis with the indicated antibody (LC3 and p62 antibody for detecting autophagy; GAPDH was used as a loading control). (B) Confocal microscopy was used to detect the formation of GFP-LC3 puncta. Original magnification, 400×. (C) Quantification of cells with >5 GFP-LC3 puncta. The data were presented as the mean ±SD from three independent experiments. (D) Apoptosis was assessed by flow cytometry with Annexin V/PI stain. (E) Cell death was assessed by LDH release into the supernatant. *p<0.05.

### OGD-induced hepatocyte autophagic apoptosis reduced by 3-MA treatment

Autophagy was reported to play double roles, protecting cells from apoptosis or inducing apoptosis depending on cellular status. To detect the role of autophagy in OGD-induced cell death, HL-7702 cells were pre-incubated with 3-Methyladenine (3-MA), an autophagy inhibitor before OGD. 3-Methyladenine (3-MA) inhibits autophagy by blocking autophagosome formation via the inhibition of phosphatidylinositol-4,5-bisphosphate 3-kinase (PI3K) [21]. Our results showed that the ratio of LC3II/I in OGD-treated HL-7702 cells was significantly inhibited and the decline in p62 expression was attenuated by 3-MA treatment (Fig. 3A). To further confirm that the OGD-induced cell death was associated with autophagy, LC3 was overexpressed in the HL-7702 cells infected by Ad-GFP-LC3 before 3-MA and OGD treatments. The punctate staining pattern of GFP-LC3 was observed. 3-MA significantly decreased the amount of OGD-induced GFP-LC3 punctate (Fig. 3B-C). Moreover, the apoptotic rate induced by OGD in HL-7702 cells measured by Annexin V/PI and supernatant LDH release was significantly reduced by 3-MA treatment (Fig. 3D-E), respectively. This demonstrates that autophagy inhibition by 3-MA prevented OGD-induced HL-7702 cell autophagy and apoptosis and that autophagy played a pro-apoptotic role in OGD-mediated hepatocyte injury.

**Figure 4.**
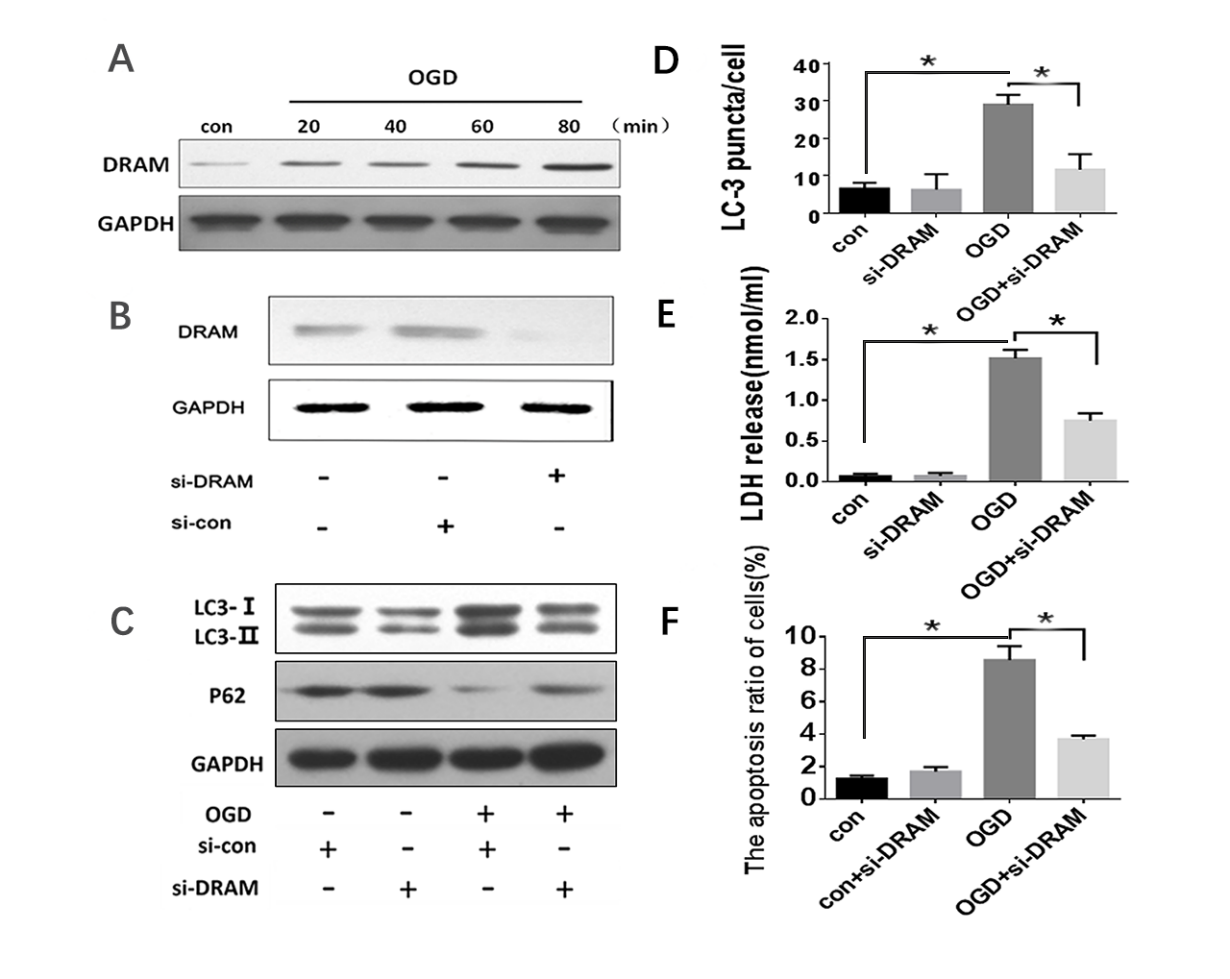
**The effects of DRAM knockdown in response to OGD-induced autophagy and apoptosis in HL-7702 cells**. Cells were transfected with siRNA-DRAM and siRNA-control and then treated with OGD for 40 minutes. (A) DRAM protein levels were detected by western blot. (B and C) Western blot analysis was used to detect autophagy levels with indicated antibody (LC3 and p62). GAPDH was used as a loading control. (D) Quantification of cells with >5 GFP-LC3 puncta. The data were presented as mean ± SD of three independent experiments. (E) Cell death was assessed by LDH release assay into the supernatant. (F) Apoptosis was assessed by flow cytometry with Annexin V/PI scan. * p<0.05.

### DRAM was a critical inducer of autophagic apoptosis in hepatocytes treated with OGD

Our previous studies have demonstrated the effects of DRAM-mediated autophagy-induced apoptosis [16, 17, 22]. Fig. 4A shows that DRAM expression levels increased in OGD-treated HL-7702 cells in a time-dependent fashion. To further confirm our hypothesis that DRAM plays an important role in OGD-induced HL-7702 cell autophagy, we knocked-down DRAM by Lipofectamine® 2000-mediated DRAM siRNA. Cells infected with DRAM siRNA obviously decreased DRAM protein expression compared to those infected with control siRNA (Fig. 4B). siRNA-mediated DRAM-knockdown partially reversed the OGD-induced LC3II/I ratio and p62 expression levels (Fig. 4C). Also, DRAM-knockdown decreased the amount of GFP-LC3 punctate induced by OGD (Fig. 4D). Moreover, LDH release and flow cytometry showed that DRAM-knockdown significantly reduced HL-7702 cell death and apoptosis induced by OGD (Fig. 4E-F). However, there was no significant change in autophagy and apoptosis with DRAM-knockdown alone. Autophagy and apoptotic levels were significantly increased in cells treated with OGD alone (Fig. 4C-F). These data indicate that DRAM contributed to OGD-induced autophagic apoptosis in normal hepatocytes.

**Figure 5.**
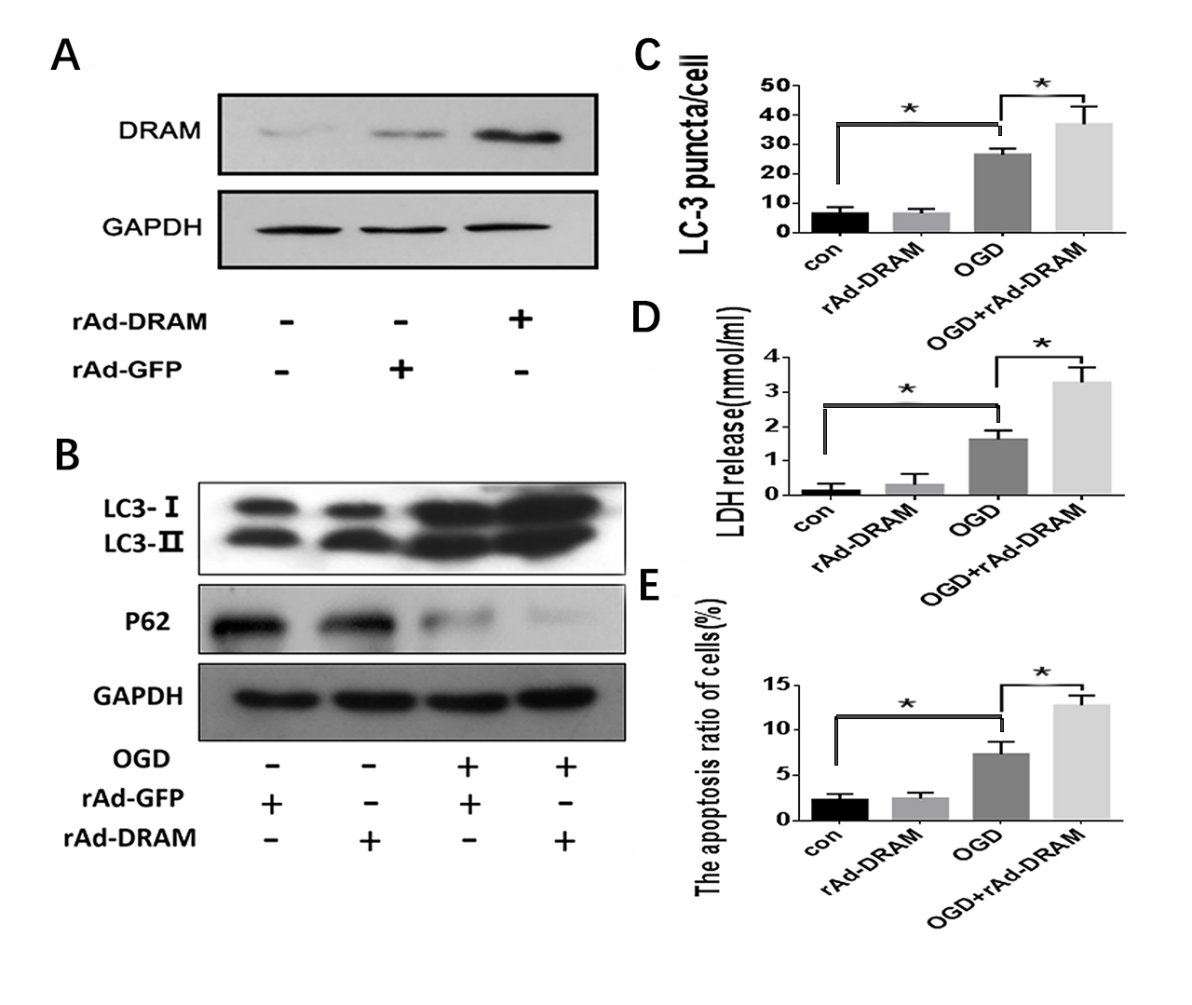
**Overexpression of DRAM enhanced OGD-induced autophagy and aggravated cell apoptosis**. HL-7702 cells were transfected with rAd-DRAM or rAd-con for 48 hours before OGD treatment. (A) The efficiency of adenovirus-mediated DRAM expression was evaluated by western blot analysis. (B) Western blot analysis was used to detect autophagy levels with the indicated antibody (LC3 and p62 antibody for detecting autophagy; GAPDH was used as a loading control). (C) Quantification of cells with >5 GFP-LC3 puncta. The data were presented as mean ± SD of three independent experiments. (D) Cell death was assessed by LDH release assay. (E) Apoptosis was assessed by Flow Cytometry with Annexin V/PI stain. *p < 0.05.

### Autophagic apoptosis aggravated by DRAM overexpression in hepatocytes treated by OGD

As above, DRAM-knockdown significantly reduced OGD-induced HL-7702 cell apoptosis. We also evaluated the effect of DRAM overexpression by rAd-DRAM infection on hepatocyte autophagic apoptosis. rAd-DRAM infection effectively increased DRAM expression level in HL-7702 cells for 48 hours (Fig. 5A). DRAM overexpression enhanced the OGD-induced LC3 II/I ratio (Fig. 5B) and the amount of GFP-LC3 punctate (Fig. 5C). As expected, OGD significantly induced HL-7702 cell apoptosis and overexpression of DRAM was significantly enhanced OGD-induced apoptosis and LDH release in HL-7702 cells compared to the cells infected with rAd-control (Fig. 5D-E). These data demonstrate that OGD-induced hepatocyte apoptosis occurs through a DRAM-dependent autophagy pathway.

### Effects of DRAM overexpression on liver function and histopathology in a mouse model of 70% hepatic ischemia

**Figure 6.**
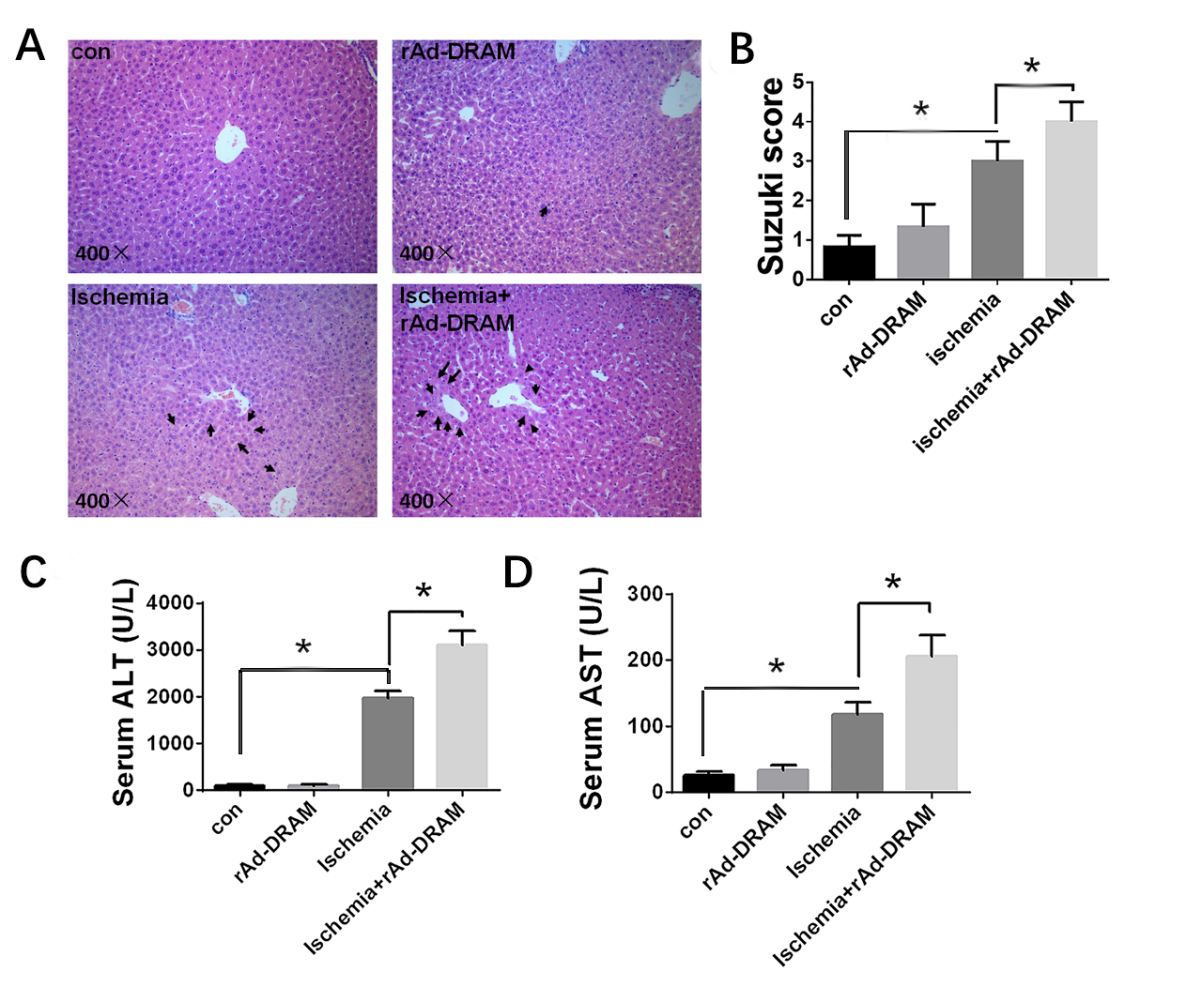
**Effects of DRAM overexpression on liver function and histopathology in a mouse liver ischemia model**. Balb/c mice were injected with rAd-DRAM or rAd-control through the tail vein 72 hours before the onset of 70% hepatic ischemia. (A) Representative histopathology of liver sections (hematoxylin-eosin) in each group. Magnification, 400×. The black arrows in the figures represent hepatocytes vacuolization. (B) The severity of liver injury assessed by the modified Suzuki classification. (c and d) Serology tests of AST and ALT in each group. Data were presented as mean ± SD. *p < 0.05.

As shown above, DRAM played an important role in OGD-induced hepatocyte injury *in vitro*. To evaluate the role of DRAM in liver injury induced by ischemia *in vivo*, we overexpressed DRAM in a mouse liver ischemia model (see ‘mice and 70% hepatic ischemia’ in Materials and Methods section). Mice were pretreated with either rAd-DRAM (5×10^10^ PFU/ml, 0.2 ml per mouse) or rAd-control (empty viral vector, 5×10^10^ PFU/ml, 0.2 ml per mouse) via tail vein injection. Partial liver ischemia surgery or sham surgery was performed at 72 hours after adenovirus injection. We compared histopathologic changes of hepatic ischemia in each group with hematoxylin and eosin (HE) staining and scored them according to Suzuki’s classification (Fig. 6A-B). The Suzuki scores quantified in congestion, vacuolation, and necrosis [20]. The ischemia + rAd-DRAM group showed significantly higher Suzuki scores than the ischemia + rAd-control group. ALT and AST in each group were measured by serologic tests (Fig. 6C-D). Significantly higher ALT and AST levels were seen in the ischemia + rAd-DRAM group compared to the ischemia+rAd-control group. Furthermore, liver transaminase and histopathology levels were significantly increased in ischemia groups compared with control groups (p < 0.05) (Fig. 6A-D).

### Effects of DRAM overexpression on hepatic autophagy and apoptosis in a mouse model with hepatic ischemia

**Figure 7.**
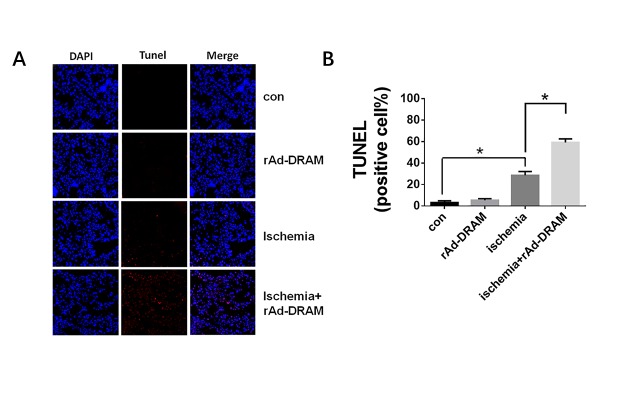
**Effect of DRAM overexpression on liver ischemia-induced cell apoptosis**. Balb/c mice were injected with rAd-DRAM or rAd-control through the tail vein 72 hours before the onset of 70% hepatic ischemia. (A) TUNEL staining results (200×), the red arrow indicates an apoptotic nucleus. (B) Statistical analysis of (A). Data were presented as mean ± SD. *p < 0.05.

**Figure 8.**
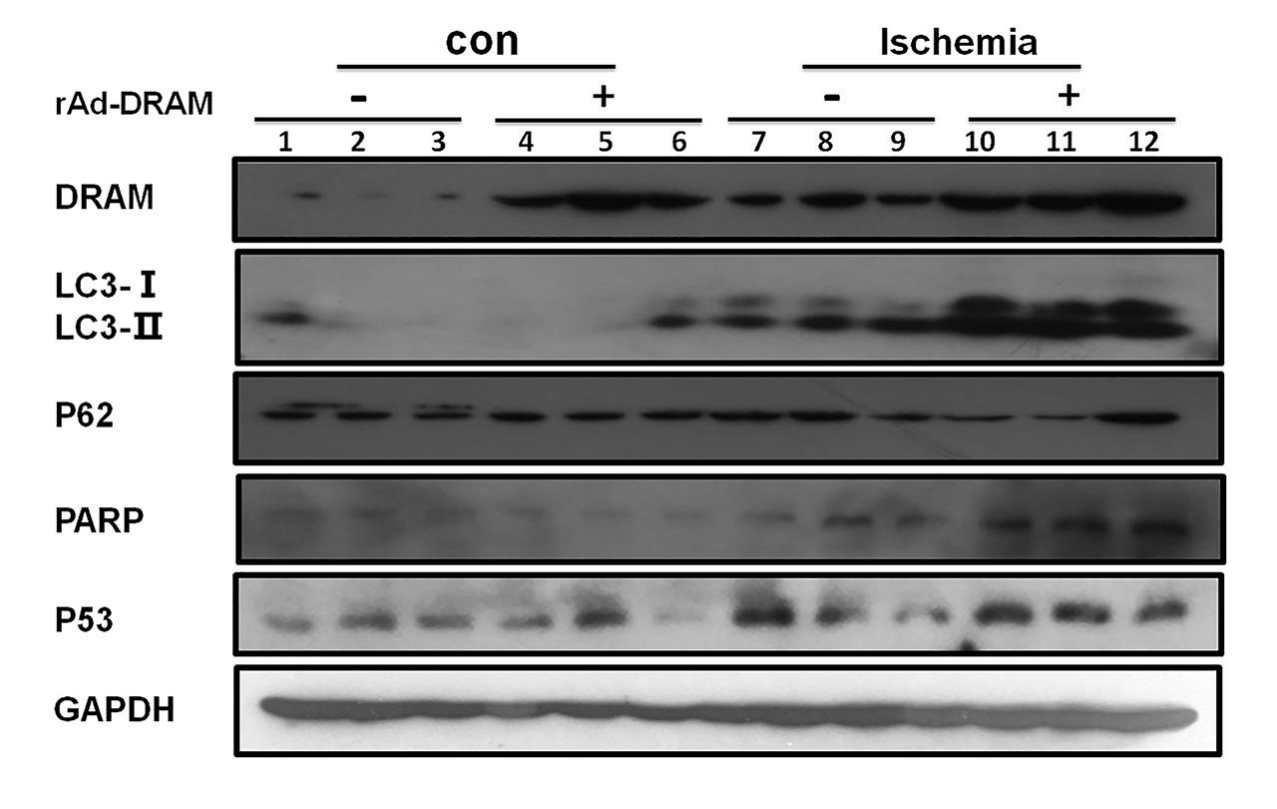
**Effects of DRAM overexpression on autophagy and apoptosis in a mouse model with 70% hepatic ischemia**. Balb/c mice were injected with rAd-DRAM or rAd-control through the tail vein 72 hours before the onset of 70% hepatic ischemia. Western blot analysis was used to detect protein levels of LC3 and p62 (markers for autophagy) and PARP and p53 (markers for apoptosis). GAPDH was used as a loading control.

Consistent with our *in vitro* study, we demonstrated above that DRAM also mediated the ischemia-induced liver injury *in vivo*. The effect of DRAM on hepatocellular autophagy and apoptosis in this mouse ischemia model remains unclear. Tunel staining was used in this model to evaluate the effect of DRAM on ischemia-induced hepatic apoptosis (Fig. 7A-B). A significant amount of apoptotic cells was seen in liver tissue with ischemia, but not in normal tissue. Overexpression of DRAM significantly increased the amount of ischemia-induced apoptotic cells (ischemia+rAd-DRAM group vs. ischemia + rAd-control group (p < 0.05)). Western blot analysis reaffirmed the pro-apoptotic properties of DRAM (Fig. 8). Significantly higher expressions of apoptotic markers (PARP and p53) were observed in the ischemia+rAd-DRAM group than that in the ischemia + rAd-control group (p<0.05). Meanwhile, of the markers for autophagy, LC3II expression was significantly increased in the ischemia + rAd-DRAM group compared to that in the ischemia + rAd-control group. Western blot analysis also showed that DRAM expression in liver tissue was increased by liver ischemia alone and rAd-DRAM. These data suggest that the animal experimental results confirmed our *in vitro* data.

## DISCUSSION

In the present study, we found that DRAM enhanced OGD and ischemia-induced damage in hepatocytes, which was closely associated with autophagic apoptosis [23].

OGD induces autophagy in different cell types such as nerve cells, cardiomyocytes and endothelial cells [24-27], while the role of OGD-induced autophagy in cell apoptosis remains controversial. Autophagy activation is increased with OGD in neuronal PC12 cells [28], and Icariin (ICA), an anti-apoptosis neuroprotective agent is able to protect PC12 cells from OGD/reperfusion-induced autophagy [29]. However, rapamycin, an immune-suppressive and anti-tumor agent, effectively prevented OGD-induced vascular endothelial cell injury by activating autophagy [30]. It has been reported that the inhibition of autophagy could facilitate the OGD-induced apoptotic process in HepG2 hepatoma cell line [31]. In contrast, we demonstrated that OGD-induced autophagy played a pro-apoptosis role in normal hepatocyte injury, we hypothesized that the difference between these cells may be due to the different properties of normal resting hepatocyte from proliferating hepatoma. Moreover, we found that inhibition of autophagy by 3-MA could rescue the harmful effect of OGD on hepatocyte. This finding is consistent with Shi’s results that OGD-activated autophagy contributes to cerebral ischemia-induced neuronal death [32].

Our current study showed that hypoxia/ischemia-induced the hepatocyte apoptosis *via* DRAM mediated autophagy. An autophagy inhibitor 3-MA treatment relieved the OGD-induced hepatocyte apoptosis. DRAM knockdown by siRNA-DRAM on hepatocyte apoptosis and autophagy exerted a similar effect as that of autophagy inhibitor (3-MA). These data indicate that inhibition of autophagy alleviated OGD-induced hepatocyte injury. Meanwhile, overexpression of DRAM by rAd-DRAM significantly activated autophagy-related proteins and enhanced the OGD and 70% liver ischemia-induced hepatocyte apoptosis and death. These results demonstrate that the inhibition of autophagy protects the liver from apoptosis, and DRAM might mediate hypoxia/ischemia-induced hepatocyte autophagic apoptosis.

Human DRAM is a highly conserved protein through evolution that can induce the accumulation of autophagosomes [33]. Previous studies have shown that p53-induced DRAM-mediated autophagy is a pro-apoptotic element [12, 34]. Our previous finding demonstrated that starvation-induced DRAM expression and DRAM-mediated autophagic apoptosis was observed in normal hepatocytes [16]. In this study, we used OGD and 70% hepatic ischemia model to investigate the functions of DRAM in liver hypoxia/ischemia injury and we found that DRAM-mediated autophagy obviously aggravated hepatocyte autophagic apoptosis *in vitro* and *in vivo*. However, the potential mechanisms of DRAM-mediated autophagy in hepatocytes with hypoxia/ischemia are complicated and requires further investigation.

Autophagic apoptosis caused increased levels of LC3-II and becline-1 [35], which is referred to as “type I Programmed cell death” or “Apoptotic Cell Death”. In this type of cell death, there is transduction signaling regulation that bridges the autophagy and apoptosis processes [35]. For instance, as a potential inducer of apoptosis, p53 also induced DRAM-mediated autophagy [12]. In the present study, overexpression of DRAM significantly activated autophagy-related proteins LC3-II in hypoxia/ischemia-treated hepatocytes while cell apoptosis rates were enhanced. In contrast, the autophagy inhibitor 3-MA or siRNA-DRAM protected hepatocytes from the OGD-induced damage. Whether other factors take part in this hypoxia/ischemia-induced autophagic cell death will require further investigation.

In summary, we demonstrated that hypoxia/ischemia increased DRAM expression in hepatocytes and the subsequent autophagy activation by DRAM was involved in hypoxia/ischemia-induced hepatic damage. Therefore, regulation of autophagy by modulating DRAM levels may be a potential therapeutic strategy for liver hypoxia/ischemia injury.
